# Genetic and Pathogenicity Diversity of *Aphanomyces euteiches* Populations From Pea-Growing Regions in France

**DOI:** 10.3389/fpls.2018.01673

**Published:** 2018-11-19

**Authors:** Anne Quillévéré-Hamard, Gwenola Le Roy, Anne Moussart, Alain Baranger, Didier Andrivon, Marie-Laure Pilet-Nayel, Christophe Le May

**Affiliations:** ^1^IGEPP, INRA, Agrocampus Ouest, Université de Rennes 1, Le Rheu, France; ^2^UMT PISOM INRA/Terres Inovia, Le Rheu, France; ^3^Terres Inovia, Thiverval Grignon, France; ^4^IGEPP, Agrocampus Ouest, INRA, Université de Rennes 1, Université Bretagne-Loire, Rennes, France

**Keywords:** *Pisum sativum*, legume crops, Aphanomyces root rot, genetic structure, host specificity

## Abstract

*Aphanomyces euteiches* is an oomycete pathogen with a broad host-range on legumes that causes devastating root rot disease in many pea-growing countries and especially in France. Genetic resistance is a promising way to manage the disease since consistent QTL controlling partial resistance have been identified in near isogenic lines of pea. However, there are still no resistant pea varieties cultivated in France. This study aimed to evaluate the phenotypic and genetic diversity of *A. euteiches* populations from the major pea-growing regions in France. A collection of 205 isolates, from soil samples collected in infested pea fields located in five French regions, was established and genotyped using 20 SSR markers. Thirteen multilocus genotypes were found among the 205 isolates which displayed a low genotypic richness (ranged from 0 to 0.333). Two main clusters of isolates were identified using PCoA and STRUCTURE, including a predominant group comprising 88% of isolates and another group representing 12% of isolates mainly from the Bourgogne region. A subset of 34 isolates, representative of the fields sampled, was phenotyped for aggressiveness on a set of resistant and susceptible varieties of four legume hosts (pea, faba bean, vetch, alfalfa). Significant differences in disease severity were found among isolates and three groups of aggressiveness comprising 16, 17, and 2 isolates, respectively, were identified using HCA analysis. A higher diversity in pathogen aggressiveness was observed among isolates from Bourgogne, which included different legumes in its crop history. Little relationship was observed between genetic clusters and pathogenicity in the subset of 34 isolates, as expected using neutral markers. This study provides useful knowledge on the current state of low to moderate diversity among *A. euteiches* populations before resistant pea varieties are grown in France. New insights and hypotheses about the major factors shaping the diversity and evolution of *A. euteiches* are also discussed.

## Introduction

*Aphanomyces euteiches* Drechsler is an oomycete pathogen of legumes, which causes the devastating root rot disease of pea (*Pisum sativum* L.) worldwide (Kraft and Pfleger, 2001). In Europe, *A. euteiches* was first observed in Norway in 1925 (Sundheim, 1972), and was reported a few years later in France (Labrousse, 1933), where it has been considered to be highly damaging in infested pea areas since 1993 (Didelot and Chaillet, 1995). The increased frequency of pea crops in French crop rotations since 1978 is believed to have favored the development of the disease in all pea-producing areas (Wicker and Rouxel, 2001). To date, the disease has been recorded in pea-growing regions such as the Bassin-Parisien, Bretagne, Rhônes-Alpes, Pyrénées, and Charente-Maritime (Wicker, 2001).

*A. euteiches* is a diploid, homothallic pathogen, i.e., having the capacity to complete its sexual cycle in the absence of any other individual (Lin and Heitman, 2007), that produces both oospores (sexual reproduction) and zoospores (asexual reproduction). Oospores can survive in the soil for more than 10 years (Papavizas and Ayers, 1974), and can resist unfavorable conditions (e.g., desiccation, freezing). *A. euteiches* was also reported to infect other legume species including alfalfa (*Medicago sativa*) (Delwiche et al., 1987), green bean (*Phaseolus vulgaris*) (Pfender and Hagedorn, 1982), faba bean (*Vicia faba*) (Lamari and Bernier, 1985) and common vetch (*Vicia sativa*) (Tsvetkova and Kotova, 1985). Currently, the main methods for managing the disease in France include avoidance of highly infested fields, which are diagnosed using an inoculum potential test, measuring disease severity on susceptible plants grown in field soil samples within a bioassay (Moussart et al., 2009), and crop rotations with non-host or resistant legume crops (Moussart et al., 2013). There are still no cultivated resistant varieties in France but consistent Quantitative Trait Loci (QTL) controlling partial resistance have been identified, validated in pea (Lavaud et al., 2015; Desgroux et al., 2016) and are being used in French pea breeding programs for the development of future resistant varieties. To support breeding for efficient resistance against *A. euteiches* populations present in French pea-growing regions more knowledge is required about the genetic diversity and the adaptive capacities of *A. euteiches* populations. A description of the state of *A. euteiches* population diversity before resistant varieties are grown is necessary to provide reference information for future comparisons with pathogenicity diversity after the development of resistant varieties.

Previous studies have described the diversity of *A. euteiches* populations worldwide. In the USA, results, obtained with dominant markers, showed high genetic diversity at the field and/or regional scales (Malvick et al., 1998; Grünwald and Hoheisel, 2006). Different genetic subpopulations were identified, which were distinguished based on the host of origin or host preference. However, all pea-infecting populations of *A. euteiches* showed significant linkage disequilibrium between markers, which suggested that selfing had played an important role in shaping the genetic structure of these populations (Grünwald and Hoheisel, 2006). In France, a preliminary study of the genetic diversity using AFLP markers found unstructured populations among 56 pea *A. euteiches* isolates collected in infested fields (unpublished data). Recently, Mieuzet et al. (2016) and Le May et al. (2018) used SSR and Sequence Related Amplified Polymorphisms (SRAP) markers, respectively, to show the absence of genetic structure among French pea isolates sampled from research disease nurseries. However, in the same study, Le May also found significant genetic structure among American isolates sampled from four American nurseries.

Prior studies also analyzed phenotypic diversity among pea-infecting *A. euteiches* isolates based on their pathogenicity on legume species and pea lines. In our study, the term “pathogenicity” included two principal characteristics generally evaluated in pathogens, including virulence and aggressiveness. Virulence refers to the ability of the pathogen to cause a susceptible response on a host plant (Parlevliet, 2002). Aggressiveness refers to the quantitative variation measured on susceptible hosts. In practice, aggressiveness can be measured through a variety of quantitative traits, including infection efficiency, latent period, spore production rate, infectious period and lesion size, expressed during the host–pathogen interaction (Sackett and Mundt, 2005). In the USA, Malvick and Percich (1998) observed field-dependent variations in pathogenicity on pea, alfalfa, and bean hosts among 114 isolates from pea fields in Minnesota, Wisconsin and Oregon. In France, Wicker et al. (2001) identified four types of pathogenicity among 91 French isolates, according to their aggressiveness on five legume species. Most of the isolates were pathogenic on at least three of the five species. Wicker and Rouxel (2001) analyzed the pathogenic variability of 88 French pea-infecting isolates compared to 21 foreign isolates on a differential set of six pea genotypes. All the French isolates belonged to one major pathotype, i.e.,virulence group (pathotype I) and a wide range of aggressiveness was observed between these isolates. Another pathotype (pathotype III), which showed lower aggressiveness toward the pea genotype MN313, was identified among American isolates.

Thus, low genetic and pathogenicity diversity among pea-infecting *A. euteiches* isolates was previously reported in France. However, these findings were obtained from collections of small to medium size isolates (< 100 individuals) partially isolated from nurseries, using mostly dominant markers that could not reveal heterozygosity on the *A. euteiches* genome, and testing the strain aggressiveness on a limited set of genotypes or legume species. However, we can hypothesize that the diversity of *A. euteiches* populations in France is wider than previously described, because pea is not grown at the same frequency and density in the different French regions and other host legume crops can be grown as intercrops. In addition, *A. euteiches* isolates analyzed in previous studies do not represent the diversity of agronomic and pedo-climatic situations existing in the different French regions grown in peas. Therefore, this study addressed the following question: what is the genetic and pathogenicity diversity of *A. euteiches* populations in pea-growing regions of France? To answer this question, a large novel collection of pea-infecting isolates from major French pea-growing regions was established, as well as a set of differential genotypes of four legume species used in crop rotations in France. The collection was characterized using co-dominant and neutral SSR markers and genetic structure of the French pathogen populations was analyzed. Pathogenicity diversity was evaluated on a subset of isolates representing the genetic diversity within the populations.

## Materials and methods

### Creation of the collection of *A. euteiches* isolates

A total of 205 *A. euteiches* isolates were obtained from soils sampled between 2011 and 2013 in 17 infested fields, distributed within eight departments corresponding to the main French pea-growing regions (Table 1). These fields belong to a network of plots for epidemic monitoring in the main production areas of pea. The choice of fields was based on the positive results of the inoculum potential tests (Moussart et al., 2009). Fifteen to twenty soil sub-samples were collected in each field at a 20–25 cm depth across the field, according to a spatial distribution in “W” (Campbell and Madden, 1990). Soils were sampled at regular intervals along the spatial pattern in “W.” The soil sub-samples from each field were mixed and 3 liters of soil per field were stored at 5°C. The baiting method used to sample isolates from each 3 liter-soil sample was adapted from Moussart et al. (2001), using the soil indexing method developed by Sherwood and Hagedorn (1958). Soils of each field were placed in pots (4 pots/field) and five plants of the pea cv. Lumina were sown in each pot (20 plants/field). A total of 68 pots was used to harvest the 205 *A.euteiches* isolates from the 17 infested fields. Four to twenty-two isolates were isolated from necrotic roots observed on 20 plants per soil, grown for 14 days, in a climatic chamber under favorable conditions to the disease (photoperiod 8–16 h, temperature 23–25°C, saturating moisture). The heterogeneous number of isolates per soil sample was due to variation in the number of symptomatic plants and level of plant infection between fields. No isolates were isolated from single-zoospore progeny since low level of polymorphism was previously reported between single-zoospore progeny and parental strains (Grünwald and Hoheisel, 2006).

**Table 1:** Origin of the 205 *Aphanomyces euteiches* isolates of the collection, collected in different French fields between 2011 and 2013.

**Region**	**French department (area code)**	**Field (population code)**	***N***	**Latitude**	**Longitude**
Bretagne	Finistère (29)	Riec/Belon (RB)	6	47°52′40.69″N	3°42′28.57″O
	Morbihan (56)	Bignan (BI)	6	47°52′51.30″N	2°44′21.70″O
Bourgogne	Côte d'Or (21)	Bretenière SO (BRO)	8	–	–
		Bretenière S1 (BR1)	16	47°14′17.68″N	5°05′39.37″E
		Bretenière S2 (BR2)	4	47°14′18.96″N	5°05′42.15″E
		Bretenière S3 (BR3)	8	47°14′34.44″N	5°05′44.90″E
Center	Eure-et-Loir (28)	Houville La Branche SO (HBO)	12	48°27′03.55″N	1°38′25.93″E
		Houville La Branche S1 (HB1)	22	48°27′23.30″N	1°38′20.98″E
		Pierres (P)	20	48°34′21.54″N	1°32′07.79″E
		Fresnay l'Evêque (F)	12	48°14′45.00″N	1°48′32.00″E
	Loiret (45)	Bleville (BL)	13	48°19′47.80″N	2°22′96.20″E
Nord-Pas-de-Calais	Nord (59)	Ennevelin SO (ENO)	21	50°31′50.12″N	3°07′45.10″E
		Ennevelin S1 (EN1)	8	50°31′50.71″N	3°08′09.90″E
Ile de France	Essonne (91)	Nainvilles Les Roches (NR)	16	48°51′96.00″N	2°49′00.00″E
		Mondeville (MO)	9	48°28′57.00″N	2°25′15.00″E
		Boigneville (BO)	11	48°32′94.90″N	2°38′72.20″E
	Seine et Marne (77)	Crisenoy (CR)	13	48°35′43.13″N	2°45′10.58″E

*N, Number of isolates sampled in each field*.

### DNA extraction and SSR genotyping

DNA was extracted from mycelium samples of the 205 *A. euteiches* isolates. Mycelial explants were grown for 6 days at 25°C on Corn Meal Agar medium (CMA). Seven to ten agar discs (3 mm diameter) per culture were then transferred to peptone-glucose broth and grown for 6 days at 25°C. Mycelial mats were vacuum-filtered on Whatman paper, rinsed three times with sterile water, and transferred to Eppendorf tubes. The harvested mycelia were lyophilized and stored at −20°C. Twenty mg of mycelium were ground with a FastPrep® grinder and extracted with the Macherey-Nagel NucleoSpin® Plant II kit (Macherey-Nagel GmbH & Co. KG, France) according to the manufacturer's recommendations. The quality and quantity of DNA were evaluated using spectrophotometry and adjusted (Nanodrop ND-100; Nanodrop Technologies) to 10 ng/μL. The extracted DNAs were then stored at −20°C.

Twenty SSR primers, previously developed to amplify *A. euteiches* DNA (Mieuzet et al., 2016), were used in this study. The PCR reaction in simplex was carried out in 10 μL containing 1X Green GoTaq®Flexi Buffer, 2 mM MgCl2, 0.2 mM each dNTP (Promega, France), 1 μM each forward and reverse primer and 0.25 μM fluorescent-labeled M13 primer (FAM, Applied Biosystem), 1 U of GoTaq Flexi DNA polymerase (final concentration) (Promega, France) and 20 ng template DNA. Volumes were adjusted to 10 μL with sterile distilled water. Amplifications were conducted on a S1000 Thermal Cycler (Bio-Rad). The cycling conditions for PCR amplification were as described in Mieuzet et al. (2016). The amplified products were separated on 1.5% agarose (Lonza) gels, stained in 1X Tris-Borate EDTA (TBE) buffer containing 1X Sybr® Safe (Invitrogen) and visualized under UV light. They were diluted 1:75 then 1:25 in sterile distilled water to decrease the signal intensity and to perform a clear analysis of peaks on GeneMapper 3.7 (Applied Biosystems) Two microliters of this dilution was then added to 10 μL HiDi formamide (Applied Biosystems) and 0.1 μl GeneScan 500 LIZ Size Standard (Applied Biosystems) then run on an ABI 3130xl Genetic Analyzer (Applied Biosystems). The presence of alleles and their sizes were assigned using GeneMapper 3.7 (Applied Biosystems). To check for the absence of genotyping error, the reference isolate RB84 and water were used as positive and negative control, respectively, in three replicates. Samples displaying non-obvious peaks or specific molecular patterns were duplicated.

### Genetic diversity analysis

SSR data were used to define MultiLocus Genotypes (MLGs) and checked for repeated MLGs. The number of repeated MLGs was identified using GENCLONE 2.0 (Arnaud-Haond and Belkhir, 2007). Genotypic evenness was evaluated using the index R = (G-1)/(N-1), with G the number of distinct multilocus genotypes and N the number of isolates (Grünwald et al., 2003). The allelic richness (Ar) was estimated using the rarefaction method implemented in POPULATIONS 1.2.32 software (Langella, 1999), which estimated the number of alleles per locus for a reduced sample size. This analysis was carried out based on the smallest population size (*n* = 4) in a first estimation. In a second analysis small-size populations (*n* < 10) were rejected and an analysis was carried out only with the smallest remaining population size (*n* = 12). Observed (Ho) and expected (He) heterozygosity were computed using GenAlEx 6.5.2 (Peakall and Smouse, 2012). MICRO-CHECKER version 2.2.3 (Van Oosterhout et al., 2004) was used to estimate the presence of null alleles at the SSR markers. Fis (Fis = 1-Ho/He) (Weir and Cockerham, 1984) was calculated using GENEPOP (Raymond and Rousset, 1995) with and without markers that displayed null alleles for each population. Clonality was assessed with the index of association (*I*_*A*_) and $\bar{r}$_*d*_ statistic, a measure of the multilocus linkage disequilibrium, calculated using Multilocus software version 3.1b (Agapow and Burt, 2001). The index of association between the scored alleles was estimated by comparing the variance of the genetic distances among alleles in the current dataset to the mean variance of 1,000 artificial re-sampled datasets. The $\bar{r}$_*d*_ statistic is much less dependent on the number of loci than the index of association (Agapow and Burt, 2001). The *I*_*A*_ and $\bar{r}$_*d*_ are expected to be zero if populations are freely recombining and greater than zero if linkage disequilibrium between alleles is maintained through selfing (clonality).

### Population structure analysis

Partition of molecular diversity among and within regions, as well as among and within populations, was studied using analysis of molecular variance (AMOVA) (Excoffier et al., 1992; Lynch and Milligan, 1994). AMOVA was performed using Arlequin 3.1 (Excoffier et al., 2005).

A principal coordinate analysis (PCoA) of the mean pairwise population genetic distance matrix was performed using the standardized genetic distance in GenALEx 6.5 (Peakall and Smouse, 2012). *A. euteiches* isolates were clustered with and without Bourgogne population using the Bayesian Clustering approach implemented in STRUCTURE 2.3.3 (Pritchard et al., 2000). The analysis was performed using 5 × 10^5^ burn-in replicates and a run length of 2 × 10^5^ Markov chain Monte Carlo (MCMC) replicates, adopting the admixed model and the correlated allele frequencies option. The number of genetic groups (*K*-value) was estimated using the model developed by Evanno et al. (2005), which provides an estimate of the posterior probability of the data for a given K, Pr (X/K). We used the height of the modal value of the distribution as an indicator of the strength of the signal detected by STRUCTURE software. Five independent runs were performed for *K* values between one and 10 in order to verify the convergence of parameter estimates. A Discriminant Analysis of Principal Components (DAPC) was also performed using the “adegenet” package of R software (Jombart et al., 2018), to confirm and describe the genetic clusters identified. Finally, a Minimum spanning network (MSN) using Nei's distance for the different populations of *A. euteiches* was calculated with the “poppr” package (Kamvar et al., 2018) from R software, version 3.2.2 (R Core Team, 2014). The interactive tool “imsn()” was used to create minimum spanning networks. Multilocus genotypes (MLG) were collapsed to multilocus haplotypes based on the minimum genetic distance at which two individuals would be considered from different clonal lineages. Haplotypes were represented by circles containing the number of isolates, and sized in proportion to haplotype frequency.

### Pathogenicity testing

#### Pathogen and plant material

The strain aggressiveness level was evaluated for 34 of the 205 *A. euteiches* isolates, including two isolates randomly sampled in each of the 17 fields used for the collection. The RB84 French reference strain (pathotype I, Moussart et al., 2007) was also used as a control.

Pathogenicity tests were conducted on eight genotypes from four leguminous species, which previously showed various levels of resistance vs. susceptibility to the RB84 strain of *A. euteiches* (Moussart et al., 2008). The plant genotypes included: pea (*Pisum sativum*) cv. Lumina (susceptible), gm. MN313 (susceptible to pathotype I isolates, but partially resistant to pathotype III isolates, Wicker and Rouxel, 2001) and gm. PI180693 (partially resistant); alfalfa (*Medicago sativa*) cv. Zenith (susceptible); vetch (*Vicia sativa*) cv. Amethyste (susceptible) and cv. Topaze (resistant); faba bean (*Vicia faba*) cv. Baraca (moderately susceptible) and cv. Melodie (resistant).

#### Pathogenicity tests and disease severity assessment

The 34 isolates were assayed in an experiment comprising eight pathogenicity tests conducted in controlled conditions (Additional File 1). Each test included four to five isolates and the RB84 strain, individually inoculated on the eight genotypes. Each test comprised four replicates of four to five plants per genotype and isolate tested. The experiment was performed twice, in 2016 and 2017, respectively, in the same climatic chamber, in order to confirm the results. A modified version of the standardized test developed for evaluating pea resistance to *A. euteiches* was used (Moussart et al., 2001). Seeds were sown in plastic pots (9^*^9^*^9.5 cm) containing unsterilized vermiculite (VERMEX, M). Faba bean seeds were soaked in water for 2 h before sowing. In each pot, five seeds of one pea, vetch or alfalfa genotype, and four seeds of one faba bean genotype were sown. Each pot constituted a replicate. Trays containing the four replicates (pots) were placed in a randomized design in a growth chamber (thermo period: 25/23°C and 16 h photoperiod). Seven days after sowing, each plant was inoculated by applying 5 ml of a zoospore suspension adjusted to 5.10^3^ spores/ml, as previously described by Moussart et al. (2001). After inoculation, the vermiculite was saturated with water to provide favorable conditions for infection. After 10 days, the plants were carefully removed from the vermiculite, the roots were washed in tap water and disease severity (DS) was scored on each plant using a 0–5 scoring scale (14): 0 = no symptoms; 1 = traces of discoloration on the roots (< 25%); 2 = discoloration of 25 to 50% of the roots; 3 = discoloration of 50 to 75% of the roots; 4 = discoloration of >75% of the roots; 5 = plant dead.

#### Statistical analysis of phenotypic data

Statistical analysis was performed using R software, version 3.2.2 (R Core Team, 2014).

To check that a given isolate shows consistent results across experiments, correlations were estimated between DS score means obtained for (i) the different isolates in the two experiments and (ii) the RB84 strain in the sixteen pathogenicity tests over the two experiments, using Pearson coefficients (*p*-value = 0.05).

DS scores were analyzed as ordinal qualitative data using a cumulative link mixed model (CLMM; “clmm” function, “ordinal” package) (Christensen, 2015). In a first analysis, the DS score was considered as the dependant variable, the isolate, legume species, or genotypes as fixed factors and the replicate and experiment as random factors. ANOVA tests using “car” and “RVAideMemoire” packages (Hervé M., 2015; Fox and Weisberg, 2016) were performed to evaluate the legume species and genotype effects (*p*-value = 0.05) in the CLMM model. In a second analysis conducted for each genotype, the DS score was considered as the dependant variable, the isolate as a fixed factor and the replicate and experiment as random factors. ANOVA tests using “car” and “RVAideMemoire” packages (Hervé M., 2015; Fox and Weisberg, 2016) were carried out to evaluate the isolate effects (*p*-value = 0.05) in the CLMM model. Least square means (LSMeans) were calculated for each isolate and genotype using the “lsmeans” function of the “lsmeans” package (Lenth and Herve, 2015). Then, for each genotype, LSMeans were compared to the value obtained for the RB84 reference isolate in all tests with a Tukey test (*p*-value = 0.001), using the “cld” function of the “MultCompView” package (Graves et al., 2015). The probability to obtain each DS scores (0 to 5) for each isolate per genotype was calculated with the “rating lsmeans” function. The probability of DS scores was comprised between 0 and 1.

Virulence is usually associated with the pathogen's capacity to infect a specific host (Van der Plank, 1968) and to multiply in the host (Casadevall and Pirofski, 2001). Virulence phenotypes were defined based on the DS score probability obtained from the CLMM for the four susceptible legume genotypes. An isolate was declared virulent on a legume species (P: pea; V: vetch; F: faba bean; A: alfalfa) when the probability of symptom appearance on the susceptible legume genotype (DS score ≥1, presence of oospores in roots; Wicker et al., 2001) was greater than 0.5.

Isolates were assigned to previously described pea pathotypes (Wicker and Rouxel, 2001), based on their level of aggressiveness on the three pea genotypes used in this study. An isolate was assigned to pathotype I when LSMean scores obtained from CLMM on the three pea genotypes were significantly different (*p*-value < 0.05), with Lumina, the most susceptible genotype, MN313, the genotype with intermediate behavior and PI180693, the most resistant genotype. An isolate was assigned to pathotype III when the LSMean score obtained on MN313 was both significantly lower than on Lumina and equal to or lower than on PI180693 (Onfroy et al., personnal comunication).

A principal component analysis (PCA; “pca” function, “FactoMineR” package) (Husson et al., 2007) was performed using DS LSmean scores for each isolate and genotype, in order to analyze the structure of *A. euteiches* isolates according to their phenotypic variability on the eight genotypes. A hierarchical clustering analysis (HCA) was then carried out to define groups of isolates using the “hclust” function of the “fastcluster” package (Müllner, 2015).

Statistical analysis was carried out in order to evaluate the differences between the DS scores of the different genetic clusters from the Bayesian analysis (STRUCTURE) on each plant genotype. DS scores were analyzed using a cumulative link mixed model (CLMM; “clm” function, “ordinal” package), in which the DS score was considered as the dependant variable, the genetic cluster and the genotypes as fixed factors and the replicate and experiment as random factor. ANOVA tests using “car” and “RVAideMemoire” packages were performed to evaluate the cluster and genotype effects (*p*-value = 0.05) in the CLMM model. In a second analysis conducted for each genotype, the DS score was considered as the dependent variable, the cluster as a fixed factor and the replicate and experiment as random factors. ANOVA tests using “car” and “RVAideMemoire” packages (Hervé M., 2015; Fox and Weisberg, 2016) were carried out to evaluate the cluster effects (*p*-value = 0.05) in the CLMM model. Least square means (LSMeans) were calculated for each cluster using the “lsmeans” function of the “lsmeans” package (Lenth and Herve, 2015). Then, for each genotype, LSMeans were compared with a Tukey test (*p*-value = 0.05), using the “cld” function of the “MultCompView” package (Graves et al., 2015).

## Results

### Genetic diversity and structure of *A. euteiches* populations

Genotyping of the 205 *A. euteiches* isolates with the 20 SSRs revealed a total of 42 alleles, with one to three alleles per SSR marker within the collection. Most of the isolates were homozygous at SSR loci. However, some isolates from the Bourgogne region displayed distinct patterns, with a lot of heterozygous loci (Additional File 2). A low level of SSR genotypic diversity was observed among isolates within fields, according to the number of distinct MLGs observed per field (*G* ≤ 3, except at BR1 and ENO; Table 2). Indeed, among the 205 isolates, GENCLONE analysis detected 19 different MLGs, including six MLGs differing from each other by one allele. Due to scoring errors, distinct MLGs belonging to the same clone can be found (Meirmans and Van Tienderen, 2004). As this was observed in this microsatellite data sets, each MLG pair presenting extremely low distance (i.e., 1–2 allelic differences) was grouped into the same multilocus lineages (Arnaud-Haond et al., 2007) resulting in a total of 13 MLG identified among the isolates in the collection (Additional File 3). MLGs 1 and 7 were the most represented, comprising 54 and 16% of the isolates studied, respectively. Genotypic richness (R) ranged from 0 (BRO, BR2, HBO, F, BO, and CR) to 0.333 (BR1). Only four fields (ENI, BR1, RB, and B1) showed an R index equal or greater than 0.20 (Table 2).

**Table 2:** SSR genotypic and genetic diversity parameters for each *Aphanomyces euteiches* population.

**Field**	**SSR genotypic diversity**			**Genetic diversity**							**Gametic desequilibrum**	
**(population) code**	***N***	***G***	***R***	**Ar (*n* = 4)**	**Ar (*n* = 12)**	**He**	**Ho**	**Na**	**Fis non-corrected**	**Fis corrected**	***I_A_***	$\bar{\text{r}}$*_d_*
RB	6	2	0.2	1.15	–	0.067	0	0	1	1	2.000***	1.000***
BI	6	2	0.2	1.05	–	0.022	0	0	1	1	–	–
BRO	8	1	0	1	–	0	0	0	–	–	–	–
BR1	16	6	0.333	1.75	1.4	0.267	0.269	2	−0.03	−0.146	11.372***	0.769***
BR2	4	1	0	1	–	0	0	0	–	–	–	–
BR3	8	2	0.14	1.84	–	0.403	0.788	0	−0.95	−0.95	1.000**	1.000***
HBO	12	1	0	1	1	0	0	0	–	–	–	–
HB1	22	2	0.048	1.14	1	0.053	0.005	3	0.78	−0.024	1.809***	0.677***
BL	13	2	0.083	1.05	1.05	0.021	0	1	1	1	–	–
P	20	3	0.105	1.11	1.05	0.048	0.003	2	0.96	1	0.214	0.114
F	12	1	0	1	1	0	0	0	–	–	–	–
ENO	21	4	0.15	1.12	1.1	0.054	0.005	2	0.93	1	0.656***	0.349***
EN1	8	3	0.286	1.09	–	0.034	0	1	1	1		
NR	16	2	0.066	1.05	1.05	0.025	0	1	1	1	–	–
MO	9	2	0.125	1.19	–	0.069	0	4	1	1	3.000***	1.000***
BO	12	1	0	1	0	0	0	0	–	–	–	–
CR	13	1	0	1.05	1.05	0.018	0	1	1	1	–	–

N, number of isolates; G, number of distinct multilocus genotype; R, genotypic richness; Ar, allelic richness corrected for sample size (n); He: expected heterozygosity without biais (Nei, 1978); Ho, observed heterozygosity; Na, number of SSR markers with null alleles; Fis, average deviation from Hardy-Weinberg proportions with (non-corrected) or without (corrected) null alleles; I_A_ index of association and ${\bar{r}}_{\text{d}}$ the standardized index of association (***p-value < 0.001,**p-value < 0.01, *p-value < 0.05,. p-value < 0.1); - missing data.

Genetic diversity indexes also showed low variability among isolates within fields, with a low level of heterozygosity and high proportion of allele fixation. *Ar* values did not exceed 1.84 alleles per population and were similar between populations except for BR1 and BR3 (Table 2). The rate of heterozygosity observed in each population was low, except for BR1 and BR3 (Ho = 0.269 and Ho = 0.788, respectively). Null alleles were present in the BR1, HB1, BL, P, EN0, EN1, NR, MO, and CR populations. The excess of heterozygosity in the two fields BR1 and BR3 was confirmed by a negative *Fis* value, whether corrected or not with null alleles, whereas complete allele fixation was observed for the other fields except for the HB1 population which showed a quasi-complete allele fixation (Table 2). A majority of the populations had significant *I*_*A*_ and $\bar{r}$_*d*_ (*p*-value < 0.01), suggesting high multi-locus linkage disequilibrium and clonal populations (Table 2).

AMOVA confirmed the lack of genetic subdivision between regions and revealed that 53% (*p*-value < 0.001) and 45% (*p*-value < 0.001) of the total genetic variance was partitioned among populations and within populations, respectively, whereas 2% (*p*-value = 0.025) was attributable to differences among regions.

**Figure 1:**
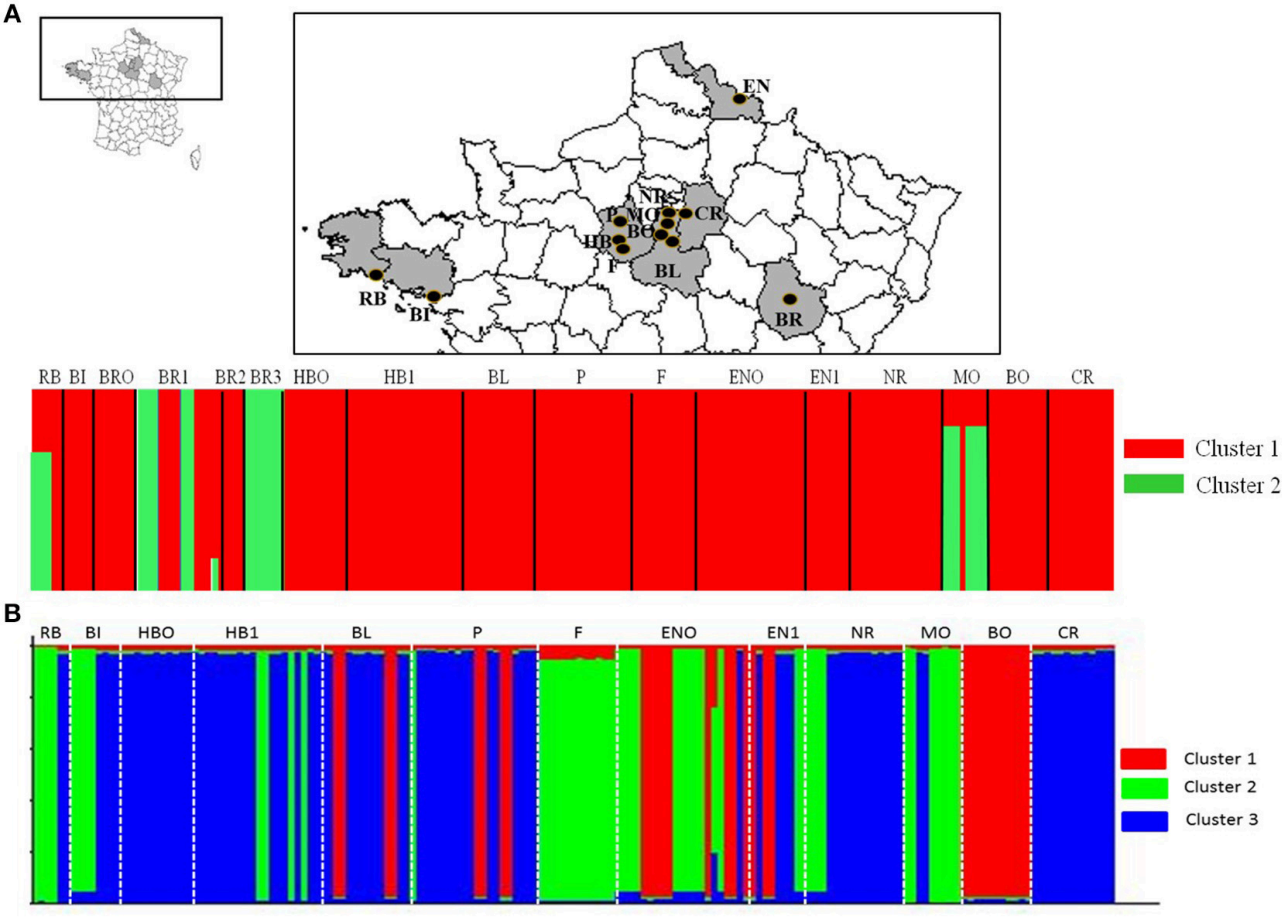
**(A)** Geographical position of fields distributed in five regions and Bayesian analysis (STRUCTURE) of the corresponding isolates, with Bourgogne (BR) data. Assignment probabilities of *A. euteiches* isolates are presented for *k* = 2 and a delta *k* = 100. Each vertical dotted line separates isolates from each of the 17 sites. **(B)** Bayesian analysis of the corresponding isolates, without Bourgogne (BR) data. Assignment probabilities of *A. euteiches* isolates are presented for *k* = 3 and a delta *k* = 1,600. Each vertical dotted line separates isolates from each of the 13 sites.

**Figure 2:**
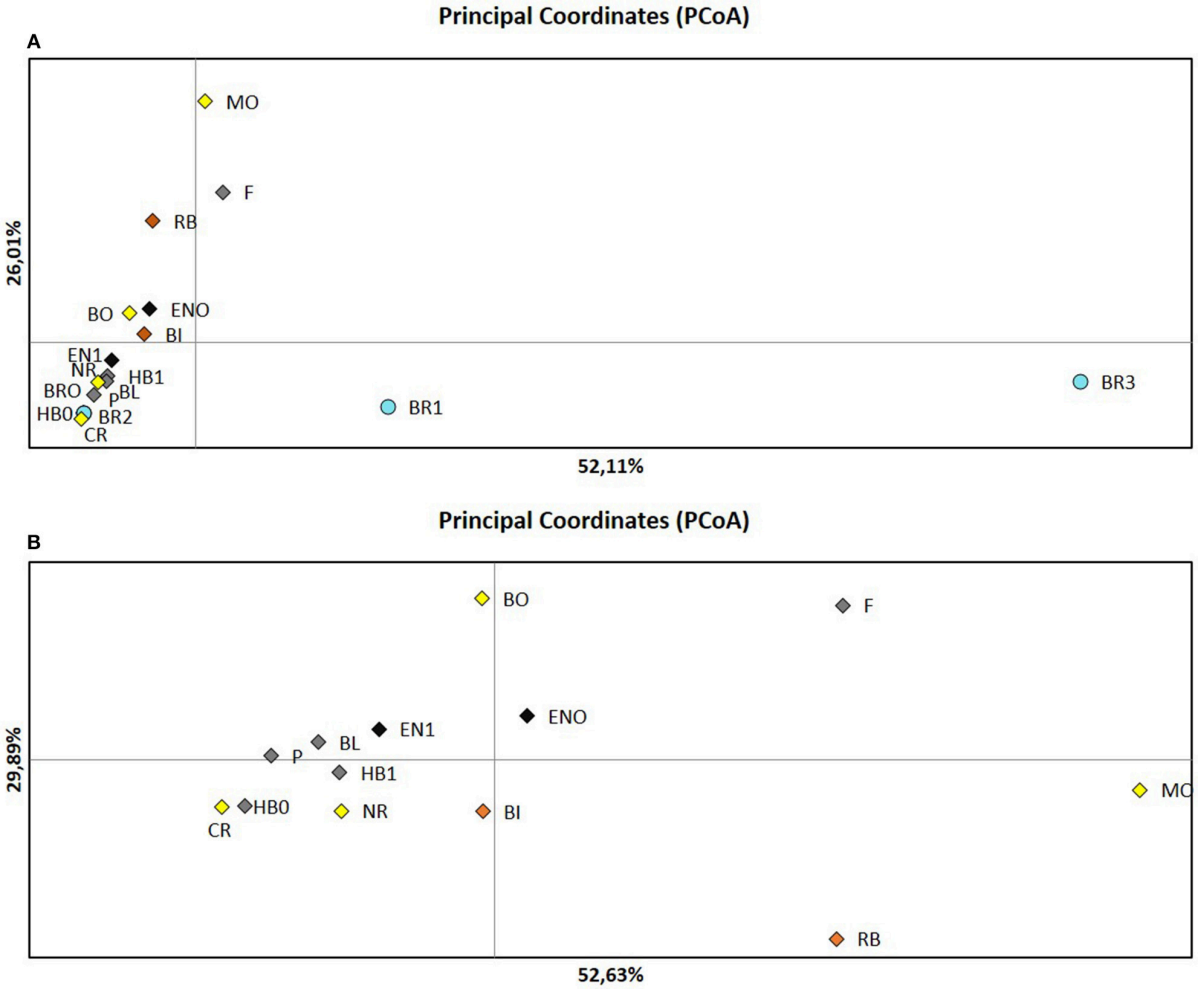
Principal Coordinates Analysis (PCoA) conducted from SSR genotypic data on the *A.euieiches* isolates of the collection. **(A)** Analysis including all the fields. **(B)** Analysis performed without Bourgogne fields. Each color corresponds to regions: yellow Ile de France, green: Center, red: Bretagne, black: Nord Pas-de-Calais, blue: Bourgogne.

STRUCTURE analysis showed a maximum log likelihood score at *K* = 2 (delta *K* = 100) (Additional File 4A), suggesting no differentiation between populations except for most populations from Bourgogne (Figure 1A, Additional File 3). Results showed that 87.8 and 12.2% of the isolates belonged to cluster 1 and cluster 2, respectively, with moderate to high inference probabilities of isolates to cluster 1 (*p* > 0.99) and cluster 2 (*p* > 0.68). Cluster 1 included isolates with homozygous genotypic patterns from the five different French regions sampled. Cluster 2 included 25 isolates, among which 14 isolates from Bourgogne (BR1, BR3) showed heterozygous genotypic profiles. PCoA analysis confirmed distinct genetic structure of isolates from Bourgogne (BR1, BR3) compared to isolates from the other fields, with the first principal axis contributing to 52.11% of the variation (Figure 2A). The distinct genetic structure of BR1 and BR3 populations was also confirmed by DAPC analysis (Additional File 5A).

Structure analysis excluding isolates from Bourgogne revealed a highest level of hierarchical sub-structure with *K* = 3 (delta *K* = 1600) (Figure 1B, Additional Files 3, 4B). However, this result is not fully consistent with the results obtained with PcoA and DAPC, which more highlighted a structure of *A. euteiches* populations according to their region of origin (Figure 2B, Additional File 5B).

**Figure 3:**
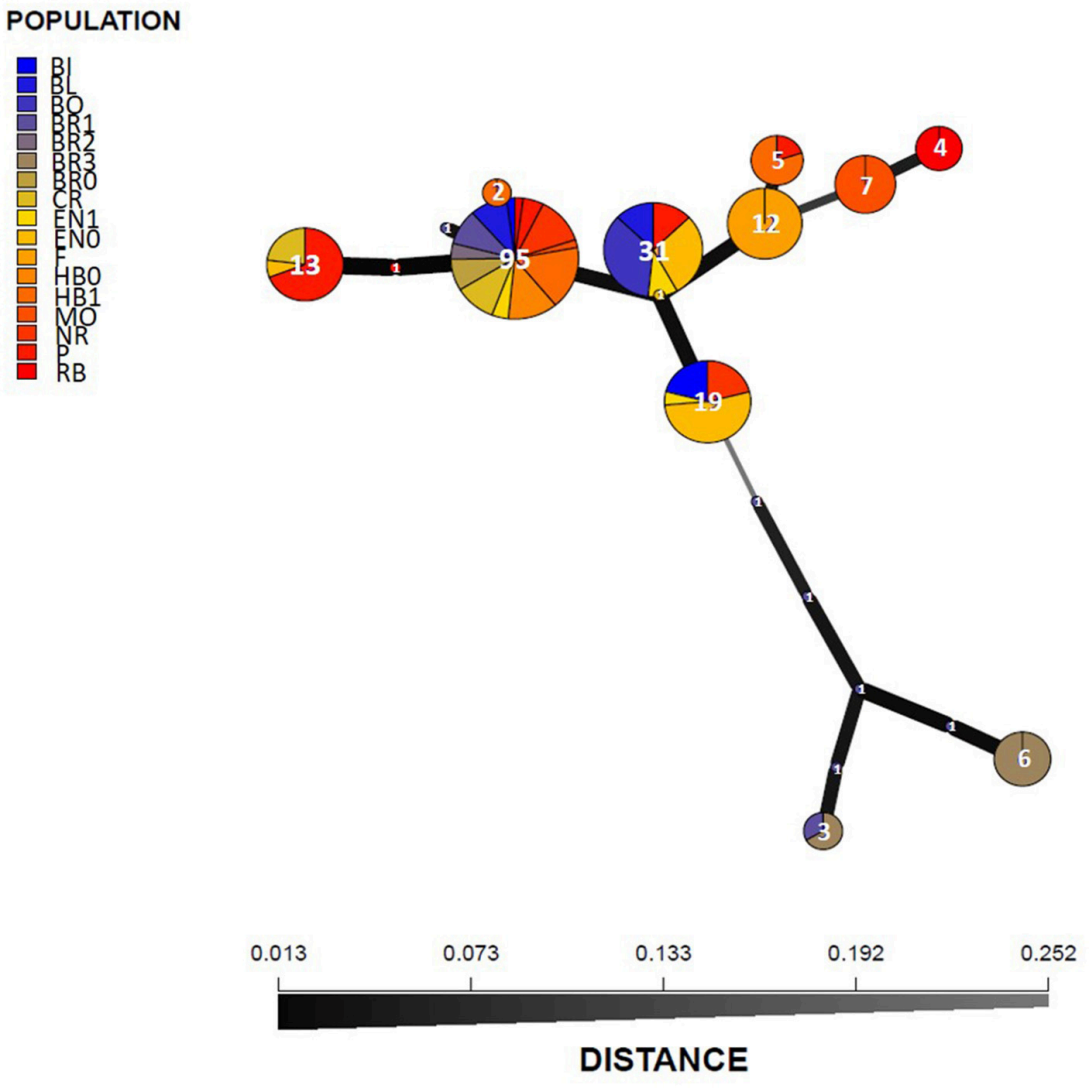
Minimum spanning network (MSN) of 19 haplotypes detected in the *A. euteiches* collection of 205 isolates. Each circle represents a unique haplotype and the colors represent the sampling fields. The circle size represents the haplotype frequency and the number of isolates was indicated in circles. Line widths and the shading represent relatedness of the haplotypes based on Nei's genetic distance.

The MSN analysis confirmed results obtained with STRUCTURE and PCoA on the entire collection, since the nineteen identified haplotypes were classified into two main groups separated by more than 0.25 Nei's genetic distance. One genetic group included isolates from Bourgogne in seven poorly represented haplotypes and the other genetic group included most of the isolates in 12 haplotypes. For the latter group there was no relationship between haplotype and geographical origin (Figure 3).

### Pathogenicity diversity of *A. euteiches* isolates

DS scores on the eight legume genotypes tested for all 34 isolates were significantly correlated between the two experiments (*r* > 0.84, *P* < 0.05). DS scores for the RB84 strain were significantly correlated between the sixteen tests in the two experiments (*r* > 0.93, *P* < 0.05).

*A. euteiches* isolates caused significantly different levels of disease severity among the tested legume species (*p*-value = 6.34.10^−15^), genotypes (*p*-value = 2.07.10^−8^) and isolates (*p*-value < 2.10^−16^). Highest levels of disease were observed on pea, followed by alfalfa, vetch and faba bean. In each legume species, our results confirmed the expected response of resistance or susceptibility of the genotypes tested. In pea, Lumina, MN313 and PI180693 presented high, intermediate and low average DS values, respectively, in response to each of the isolates tested (Table 3).

**Table 3:** Disease severity (DS) LSMean values obtained from CLMM analysis of DS ratings on roots of eight genotypes of four different legume species, in response to inoculation with 34 *A. euteiches* isolates from the collection (two isolates per field).

**Species**	**Pea**			**Vetch**		**Faba bean**		**Alfafa**	**Virulence phenotype**	**Pathotype**	**MLG**
**Isolates**	**LSMeans Lumina**	**LSMeans MN313**	**LSMeans PI180693**	**LSMeans Amethyste**	**LSMeans Topaze**	**LSMeans Baraca**	**LSMeans Melodie**	**LSMeans Zenith**		
RB84	12.328	6.813	2.236	4.119	−3.785	1.111	−2.143	1.274	PVFA	**I**	**3**
RB3	5.892	4.830	−0.537***	−0.391***	−1.729	−1.861***	−5.595	−1.150***	PVFA	**I**	**3**
RB5	8.316	5.616	1.089	0.542***	−3.856	−1.899***	−5.615	−0.071	PVFA	**I**	**1**
BI1	7.285	6.764	0.614	1.612	−4.255	−2.261***	−3.853	0.067	PVFA	**I**	**4**
BI6	8.316	7.845	1.292	1.565	−4.652	−1.655	−3.294	0.168	PVFA	**I**	**1**
BRO-2	−1.013	−3.535***	−4.583***	−9.432***	−2.320	−5.620***	−5.623	−5.468***	P	**I**	**1**
BRO-6	0.444	−1.891***	−2.775***	−7.566***	−1.547***	−5.535***	−5.603	−4.034***	P	**I**	**1**
BR1-2	2.452	3.888	−0.753***	1.121	−3.426	0.494	−2.671	2.258	PVFA	**I**	**12**
BR1-3	5.396	5.975	−0.762***	−0.654***	−2.977	0.649	−1.644	1.650	PVFA	**I**	**11**
BR2-1	7.105	5.752	0.994	−3.603***	−5.411	−1.829***	−3.510	−0.027	PVFA	**I**	**1**
BR2-4	9.035	6.675	0.923	−6.639***	−4.555	−2.508***	−5.656	−2.495***	PFA	**I**	**1**
BR3-1	5.547	5.901	−1.939***	3.124	−4.271	1.175	−1.665	1.804	PVFA	**I**	**11**
BR3-5	5.749	5.978	−1.003***	−0.949***	−3.497	0.707	−0.765	0.009	PVFA	**I**	**10**
HBO-1	8.175	4.128	−0.130	−1.640***	−4.317	−1.401	−4.028	1.939	PVFA	**I**	**1**
HBO-9	9.012	4.981	1.829	1.991	−3.003	−0.018	−2.898	−0.617	PVFA	**I**	**1**
HB1-3	7.536	6.546	−0.954***	−1.058***	−4.687	−1.396	−3.355	−1.135	PVFA	**I**	**1**
HB1-14	8.982	5.966	4.200	0.919	−5.411	−0.305	−1.313	0.560	PVFA	**I**	**1**
BL5	8.941	5.617	2.057	3.666	−3.294	1.002	−3.260	0.485	PVFA	**I**	**1**
BL8	9.012	4.371	1.713	1.414	−3.679	−0.575	−3.826	0.969	PVFA	**I**	**1**
P8	8.292	5.737	0.242	0.969	−4.688	−0.978	−2.772	0.181	PVFA	**I**	**1**
P14	8.966	4.687	0.610	0.919	−4.688	−0.365	−3.547	−0.357	PVFA	**I**	**7**
F3	9.035	6.081	3.466	2.439	−5.447	0.377	−1.589	−0.264	PVFA	**I**	**8**
F10	8.959	7.125	2.641	2.133	−4.634	0.339	−1.074	−0.123	PVFA	**I**	**8**
ENO-12	8.975	7.479	1.317	2.298	−3.954	0.634	−0.850	1.663	PVFA	**I**	**4**
ENO-16	8.064	5.592	1.121	2.466	−3.570	0.211	−2.630	2.494	PVFA	**I**	**5**
EN1-3	8.175	7.312	2.270	−8.745***	−3.109	−0.861	−3.242	1.825	PFA	**I**	**7**
EN1-5	9.035	6.196	0.451	1.633	−3.131	0.495	−2.399	0.582	PVFA	**I**	**1**
NR8	9.012	4.660	1.087	1.979	−4.707	−1.776***	−2.198	−0.240	PVFA	**I**	**1**
NR14	8.982	6.262	2.903	2.411	−3.952	0.605	−2.097	0.095	PVFA	**I**	**1**
MO1	7.853	6.783	1.920	0.843	−4.254	−1.298	−4.846	0.510	PVFA	**I**	**1**
MO5	9.006	5.584	2.487	0.558***	−5.395	−1.065	−4.914	0.003	PVFA	**I**	**6**
BO5	7.291	5.211	1.982	−11.334***	−4.271	−2.422***	−2.769	−0.673	PFA	**I**	**7**
BO13	7.105	5.932	2.507	−10.555***	−3.698	−1.906***	−3.890	−1.883***	PFA	**I**	**7**
CR5	9.035	4.925	0.390	−1.097***	−5.410	−0.168	−2.871	0.770	PVFA	**I**	**1**
CR9	8.989	6.161	1.636	1.427	−4.688	0.252	−2.435	0.515	PVFA	**I**	**1**
Mean	7.580	5.370	0.873	−0.672	−4.008	−0.847	−3.155	0.037		
Standard Deviation	2.556	2.234	1.780	4.211	0.986	1.622	1.432	1.640		

LSMean scores highlighted in gray with the isolates tested are significantly different from the LSMean score obtained with the RB84 reference strain for the same genotype (***p-value < 0.001). A Virulence phenotype is defined on pea (P), vetch (V), faba bean (F) and alfalfa (A) when the probability of DS score ≥ 1 on the four susceptible legumes (Pea: Lumina; Vetch: Amethyste; Faba bean: Baraca; Alfalfa: Zenith) is equal or higher than 0.5, according to the CLMM analysis. Pathotype I was attributed for all the isolates since LSMeans scores between the three pea genotypes were significantly different (p-value < 0.05), with Lumina, MN313 and PI180693 being the susceptible, intermediate and partially resistant genotypes, respectively (Additional File 8). MLGs (Multilocus groups) are indicated according to the genetic diversity analysis conducted in this study.

Significant DS differences were observed between several isolates of the collection and RB84, on the pea genotype PI180693, the vetch genotype Améthyste and the faba bean genotype Baraca (*p*-value < 0.0049) (Table 3). In addition, some isolates had the same effects on different genotypes (low variance) and other had different effects (high variance) depending on the French regions and the plant genotype. Isolates from Bourgogne showed the highest DS variance on almost all the genotypes tested. The vetch genotype Topaze, as well as the faba bean genotype Baraca and mostly Melodie, had the most highly diverse DS scores in response to the isolates sampled from the different regions (Additional File 6). Twenty eight isolates were virulent on the four susceptible legume genotypes (PVFA virulence phenotype profile). Four isolates (BR2-4, EN1-3, BO5, and BO13) were virulent on all the legumes tested but vetch (PFA profile) and two isolates from Bourgogne (BRO-2 and BRO-6) were virulent only on pea (P profile) (Table 3, Additional File 7). All 34 isolates were assigned to pea pathotype I (Table 3, Additional File 8).

**Figure 4:**
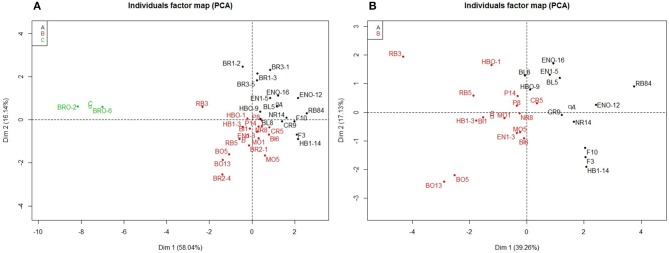
PCA of LSMean disease severity data obtained on eight legume genotypes in response to inoculation with 34 isolates of *A.euieiches*. **(A)** Analysis included all the fields sites. **(B)** Analysis was performed without Bourgogne field sites. Each color correspond to group from the hierarchical clustering ascendant analysis: black, field sites from group A, red: field sites from group B and green, field sites from group C.

Hierarchical clustering and PCA identified three clusters of pathogenicity (cluster A: 16 isolates, cluster B: 17 isolates, and cluster C: 2 isolates) among the 34 isolates (Figure 4A). Cluster C included two isolates from Bourgogne (BRO-2, BRO-6), isolated from a different field than those identified in genetic group 2 (BR1, BR3). The first PCA axis (*R*2 = 58.04%) separated cluster C from clusters A and B (Figure 4A). Hierarchical clustering on the PCA analysis performed without isolates from Bourgogne separated isolates belonging to clusters A and B regardless of their region of origin, with a first PCA axis accounting for 39.26% of the total phenotypic variability (Figure 4B). Isolates belonging to cluster A included the reference isolate RB84 and showed a PVFA virulence phenotype profile. Isolates belonging to cluster B displayed PVFA and PFA profiles and the two isolates belonging to cluster C displayed a P virulence phenotype profile (Table 3).

*A. euteiches* isolates caused significantly different levels of disease severity among the genetic cluster and genotypes (*p*-value < 2.10^−16^). On each plant genotype, significant differences (*p*-value < 0.05) were observed between LSMeans of DS scores of two out of the three genetic clusters obtained from the Bayesian analysis (STRUCTURE), except on the vetch genotypes Amethyste and Topaze. On Amethyste, DS Score LSMeans were significantly different between the three genetic clusters, whereas on Topaze, these were not different between clusters (Additional File 9).

## Discussion

*A. euteiches* is a major devastating disease of spring pea in France. Intensification of spring crops and short rotations resulted in the significant development of the disease. This study is the first report analyzing both the genetic and pathogenicity diversity of *A. euteiches* populations from French pea-growing regions using co-dominant markers. This study gives insights into (i) the genetic diversity of French *A. euteiches* populations at the regional scale, (ii) the phenotypic diversity of isolates for pathogenicity on different legume hosts, and (iii) the relationship between genotypic and phenotypic group.

### Most french *A. euteiches* populations have low genetic structure and diversity

Based on the genetic polymorphism of SSR markers, this study shows that the genetic structure of French *A. euteiches* populations is low and not related to geographical origin. A low level of genetic diversity in *A. euteiches* populations was observed between the different locations in France, as shown by the moderate number of multilocus genotypes identified (13 different MLGs within 205 isolates) including a predominant MLG (MLG1) shared by 54% of the isolates studied. This result is consistent with previous studies conducted on French and American *A. euteiches* populations, using codominant or dominant markers (Malvick and Percich, 1998; Wicker, 2001; Grünwald and Hoheisel, 2006; Mieuzet et al., 2016; Le May et al., 2018). In this study, the similar level of differentiation observed within and among populations also confirmed the absence of a clear genetic structure, as previously reported (Malvick and Percich, 1998; Wicker et al., 2001; Grünwald and Hoheisel, 2006). The absence of resistance level toward the disease and the deployment of the same pea susceptible cultivars across several geographic area in France limit selection by host. This could explain the low level of neutral genetic differentiation effects. Moreover, founder effect which can lead to reduce the genetic variation within the *A. euteiches* populations may not occur due to the production of a large number of oospores at each cropping season and the ability of oospores to be conserved in the soil for many years. The high level of linkage disequilibrium observed suggests clonal reproduction in the populations studied, in line with the low level of genotypic diversity observed. These results are consistent with the homothallic mode of reproduction of the pathogen and its lower dissemination in the soil than that of airborne pathogens in the air. These *A. euteiches* life traits contribute to limit genetic mixing and maintain highly inbred pathogen populations.

However, *A. euteiches* populations from Bourgogne appeared to have unique characteristics compared to the other locations sampled in this study. Indeed, some *A. euteiches* isolates from Bourgogne showed a specific molecular pattern, with a high level of heterozygosity, which contributed to the high level of genetic diversity observed in this region. SSR markers used in our study allowed heterozygosity to be revealed in populations from Bourgogne, whereas no previous study using dominant markers could be able to reveal such genetic profiles. An excess of heterozygous genotypes was observed for BR1 and BR3 populations, which clustered separately from the other populations in the analysis and DAPC analysis. Our results are consistent with those of Grünwald and Hoheisel (2006), which suggested that population structure of the homothallic *A. euteiches* pathogen is mostly determined by regular selfing, but also occasional recombination, indicating a mixed mode of reproduction in *A. euteiches* populations. This complex pattern of sexuality was identified in other oomycetes (Francis and Stclair, 1993; Whisson et al., 1994), suggesting that outcrossing could occur even in pathogen populations with a high level of selfing. Indeed, Grünwald and Hoheisel (2006) suggested that outcrossing and migration occurred, albeit rarely, and contributed to the genetic diversity and differentiation observed in *A. euteiches* populations sampled from two fields in Oregon and Washington in the USA.

In our study, heterozygous isolates were obtained from fields in the Bourgogne region, which included legumes (faba bean, vetch, and alfalfa) other than pea in their cropping history, in contrast to all the other fields sampled, which only had a history of pea production. We could thus hypothesize that the occurrence of these isolates may result from outcrossing between genetically distinct isolates adapted to different legumes. *A. euteiches* is reported to attack other legume species including common bean, broad bean, faba bean, clover, and alfalfa (Pfender and Hagedorn, 1982; Lamari and Bernier, 1985; Tivoli et al., 2006; Moussart et al., 2008). In some regions of the United States, where pea and alfalfa crops were frequently included in cropping systems, populations of pea-infecting *A. euteiches* showed differentiation into sub-populations with differences in genotypes and virulence toward pea and alfalfa (Holub et al., 1991; Malvick et al., 1998, 2009; Malvick and Grau, 2001). To investigate the presence of such distinct isolate genotypes, influenced by their host of origin in France, it would thus be necessary to increase the number of isolates sampled from the Bourgogne fields studied and from other fields with various legumes in their crop histories.

### Pathogenicity diversity of french *A. euteiches* populations depends on plant host and genotypes

Low to moderate pathogenicity diversity and structure was observed among the 34 isolates sampled in the collection from the different locations studied, depending on isolates and plant hosts and genotypes. Most isolates showed high aggressiveness on pea and virulence on vetch, faba bean and alfalfa (PVFA) with variable aggressiveness. Moreover, the level of aggressiveness of the isolates recorded on pea differential set used in this study indicated that all 34 isolates belong to pathotype I. Because of the heaviness of pathological tests and low genetic structure of populations studied, sub-samples of two isolates per field population were chosen to have a good geographic representation of the aggressiveness of French populations. The pathogenicity diversity observed within the French *A. euteiches* populations studied is consistent with previous studies. Using 91 pea-infecting isolates of *A. euteiches*, Wicker et al. (2001) reported that French isolates were able to infect a wide range of legume species with a low diversity of aggressiveness on pea genotypes and a moderate diversity of aggressiveness on vetch and alfalfa. The prevalence of pea crops in France since the 1980s and the susceptibility to *A. euteiches* of the pea cultivars used by the growers may explain the high aggressiveness and small variation of pathogenicity on pea of most of the French isolates tested. Moussart et al. (2008) reported a lower variability of disease response to *A. euteiches* infection in pea than in vetch and faba bean, with most varieties showing high level of susceptibility.

In this study, a poor relationship between genetic clusters and pathogenicity was observed in the subset of 34 isolates analyzed. Despite the low isolate-sample size used in this study to compare genotypic and pathogenicity diversity, the type of markers used could explain the lack of relationship observed between genetic and phenotypic structure of *A. euteiches* populations. Indeed, the neutral nature of SSR markers would probably not allow the *A. euteiches* isolates to be genotyped in genomic regions under selection. Similar results were obtained previously using RAPD or AFLP markers. Malvick and Percich (1998) did not find any correlation between pathogenic and genetic diversity in four American populations of *A. euteiches* from Minnesota, Wisconsin and Oregon, despite pathogenic variation being observed among the populations on a differential pea set. Grünwald and Hoheisel (2006) reported discrepancies between the levels of genetic and pathogenic diversity observed in two *A. euteiches* populations baited on pea from two fields located in Athena, Oregon and Mount Vernon, Washington. The use of Next Generation Sequencing methodologies to sequence the genome of *A. euteiches* isolates (Madoui et al., 2007; Gaulin et al., 2008) and further knowledge of pathogen effectors (Ramirez-Garces et al., 2016) would help to decipher the relationship between genetic and pathogenic diversity in *A. euteiches* populations.

## Conclusion

A low level of genetic and phenotypic diversity among French *A. euteiches* populations was observed in this study, based on the analysis of a collection of 205 isolates sampled from the major pea-growing regions in France. However, significant differences in aggressiveness were observed between several isolates on some genotypes. In addition, some isolates originating from fields where other legumes besides pea have been grown, also showed a distinct genetic structure with heterozygous genotypic patterns. This could suggest that crosses may have occurred between isolates which may have evolved on different hosts. Up till now, French *A. euteiches* populations have been subjected to limited selective pressure from hosts, since the diversification of legume species in rotation was restricted and no pea resistant varieties have yet been cultivated in France. Breeding for resistance is making progress, especially due to cumulating stable resistance QTL which were identified and recently confirmed in germplasm (Desgroux et al., 2016; Lavaud et al., 2016). Future partially resistant pea varieties and the increasing diversification of cropping systems with host legumes will likely modify selection pressures on the pathogen populations and thus their genetic and pathogenic structures, as suggested in this study from some isolates. A better understanding and management of the evolutionary forces affecting *A. euteiches* populations will thus be required, in order to develop integrated control strategies for the durable management of Aphanomyces root rot disease.

## Author contributions

AQ-H and GL generated the phenotypic and genotypic data. AQ-H and CL performed the statistical and genetic analyses and drafted the manuscript. AM contributed to the establishment of the collection. AM, AB, and DA provided scientific expertise on the conception of the study. CL and M-LP-N coordinated and supervised the manuscript writing and the experiments. All the authors approved the final draft of the manuscript.

### Conflict of interest statement

The authors declare that the research was conducted in the absence of any commercial or financial relationships that could be construed as a potential conflict of interest. The reviewer AG-F and handling Editor declared their shared affiliation.
