# Supplementary material for: Genetic and Pathogenicity Diversity of Aphanomyces euteiches Populations From Pea-Growing Regions in France
Source: Front Plant Sci. 2018 Nov 19;9:1673. doi: 10.3389/fpls.2018.01673 (PMC6252352; doi:10.3389/fpls.2018.01673)
Supplement: Supplementary file 2 [file Table_2.DOCX]

**Additional file 3.** Distribution of the 13 multilocus genotypes (MLGs) within and between sites. ^a, b^ The cluster number is indicated according to the Structure analysis with or without Bretenieres populations (Figure 1, Additional file X)

| **Population code** | **MLG** | **n** | **cluster assignment ^a^** | **cluster assignment ^b^** |
| --- | --- | --- | --- | --- |
|  |  |  | **delta k=100** | **without Bretenière** |
|  |  |  |  | **delta k= 1600** |
| RB | MLG1 | 2 | 1 | 3 |
|  | MLG3 | 4 | 2 | 2 |
| BI | MLG1 | 2 | 1 | 3 |
|  | MLG4 | 4 | 1 | 2 |
| BRO | MLG1 | 8 | 1 | - |
| BR1 | MLG1 | 9 | 1 | - |
|  | MLG2 | 1 | 1 | - |
|  | MLG10 | 2 | 2 | - |
|  | MLG11 | 2 | 2 | - |
|  | MLG12 | 1 | 2 | - |
|  | MLG13 | 1 | 2 | - |
| BR2 | MLG1 | 4 | 1 | - |
| BR3 | MLG10 | 6 | 2 | - |
|  | MLG11 | 2 | 2 | - |
| HBO | MLG1 | 12 | 1 | 3 |
| HB1 | MLG1 | 18 | 1 | 3 |
|  | MLG9 | 4 | 1 | 2 |
| P | MLG1 | 15 | 1 | 3 |
|  | MLG7 | 4 | 1 | 1 |
|  | MLG9 | 1 | 1 | 2 |
| F | MLG8 | 12 | 1 | 2 |
| BL | MLG1 | 9 | 1 | 3 |
|  | MLG7 | 4 | 1 | 1 |
| ENO | MLG1 | 1 | 1 | 3 |
|  | MLG4 | 10 | 1 | 2 |
|  | MLG5 | 1 | 1 | 2 |
|  | MLG7 | 9 | 1 | 1 |
| EN1 | MLG1 | 4 | 1 | 3 |
|  | MLG4 | 1 | 1 | 2 |
|  | MLG7 | 3 | 1 | 1 |
| NR | MLG1 | 12 | 1 | 3 |
|  | MLG4 | 4 | 1 | 2 |
| MO | MLG1 | 3 | 1&2 | 2&3 |
|  | MLG6 | 6 | 1 | 2 |
| BO | MLG7 | 11 | 1 | 1 |
| CR | MLG1 | 13 | 1 | 3 |

Population codes are according to Table 1. MLGs were identified using 20 SSR markers among the 205 French *A. euteiches* isolates of the collection. MLG number (MLG), number of isolates (n) and cluster assignment with all the data and without Bretenière obtained with STRUCTURE.
