# Supplementary material for: Genetic and Pathogenicity Diversity of Aphanomyces euteiches Populations From Pea-Growing Regions in France
Source: Front Plant Sci. 2018 Nov 19;9:1673. doi: 10.3389/fpls.2018.01673 (PMC6252352; doi:10.3389/fpls.2018.01673)
Supplement: Supplementary file 3 [file Table_3.DOCX]

**Additional file 8.** LSMeans of disease severity on the three pea genotypes studied in response to inoculation with 34 *A. euteiches* isolates

|  | Lumina | |  | MN313 | |  | PI180693 | |  |
| --- | --- | --- | --- | --- | --- | --- | --- | --- | --- |
|  | LSM | SD |  | LSM | SD |  | LSM | SD |  |
| RB84 | 10,2669 | 0,3208 | a | 6,8845 | 0,2913 | b | 3,8372 | 0,2664 | c |
| RB3 | 7,4682 | 0,2693 | a | 4,0858 | 0,2562 | b | 1,0384 | 0,2363 | c |
| RB5 | 8,9058 | 0,2897 | a | 5,5234 | 0,2657 | b | 2,4760 | 0,2415 | c |
| BI1 | 8,8760 | 0,2916 | a | 5,4936 | 0,2687 | b | 2,4463 | 0,2435 | c |
| BI6 | 9,7636 | 0,3112 | a | 6,3812 | 0,2848 | b | 3,3339 | 0,2593 | c |
| BR0-2 | 1,3141 | 0,2090 | a | -2,0683 | 0,2071 | b | -5,1157 | 0,2335 | c |
| BR0-6 | 3,0008 | 0,2207 | a | -0,3816 | 0,2162 | b | -3,4289 | 0,2318 | c |
| BR1-2 | 5,7363 | 0,2815 | a | 2,3539 | 0,2691 | b | -0,6934 | 0,2671 | c |
| BR1-3 | 7,4651 | 0,2588 | a | 4,0827 | 0,2429 | b | 1,0353 | 0,2233 | c |
| BR2-1 | 8,6981 | 0,2890 | a | 5,3157 | 0,2672 | b | 2,2683 | 0,2432 | c |
| BR2-4 | 9,1746 | 0,2921 | a | 5,7922 | 0,2666 | b | 2,7449 | 0,2410 | c |
| BR3-1 | 6,9682 | 0,2672 | a | 3,5858 | 0,2559 | b | 0,5385 | 0,2409 | c |
| BR3-5 | 7,4767 | 0,2685 | a | 4,0943 | 0,2546 | b | 1,0469 | 0,2366 | c |
| HB0-1 | 8,2281 | 0,3415 | a | 4,8457 | 0,3255 | b | 1,7983 | 0,3087 | c |
| HB0-9 | 9,1872 | 0,2975 | a | 5,8048 | 0,2728 | b | 2,7574 | 0,2477 | c |
| HB1-3 | 8,5126 | 0,3070 | a | 5,1302 | 0,2889 | b | 2,0828 | 0,2696 | c |
| HB1-14 | 10,3549 | 0,3394 | a | 6,9725 | 0,3124 | b | 3,9252 | 0,2867 | c |
| BL5 | 9,3201 | 0,3007 | a | 5,9377 | 0,2740 | b | 2,8903 | 0,2501 | c |
| BL8 | 8,9663 | 0,2910 | a | 5,5839 | 0,2662 | b | 2,5366 | 0,2426 | c |
| P8 | 8,5343 | 0,2866 | a | 5,1519 | 0,2658 | b | 2,1046 | 0,2425 | c |
| P14 | 8,5606 | 0,2867 | a | 5,1782 | 0,2649 | b | 2,1308 | 0,2422 | c |
| F3 | 10,0237 | 0,3293 | a | 6,6413 | 0,3026 | b | 3,5940 | 0,2786 | c |
| F10 | 10,1315 | 0,3184 | a | 6,7490 | 0,2893 | b | 3,7017 | 0,2645 | c |
| EN0-12 | 9,7391 | 0,3118 | a | 6,3567 | 0,2843 | b | 3,3094 | 0,2604 | c |
| EN0-16 | 9,0317 | 0,3684 | a | 5,6493 | 0,3488 | b | 2,6020 | 0,3304 | c |
| EN1-3 | 10,2375 | 0,4001 | a | 6,8551 | 0,3769 | b | 3,8078 | 0,3585 | c |
| EN1-5 | 8,8280 | 0,2917 | a | 5,4456 | 0,2690 | b | 2,3983 | 0,2452 | c |
| NR8 | 8,7744 | 0,2880 | a | 5,3920 | 0,2648 | b | 2,3447 | 0,2412 | c |
| NR14 | 9,8027 | 0,3105 | a | 6,4203 | 0,2818 | b | 3,3730 | 0,2580 | c |
| M01 | 9,6018 | 0,3023 | a | 6,2194 | 0,2746 | b | 3,1721 | 0,2494 | c |
| M05 | 9,4792 | 0,3003 | a | 6,0968 | 0,2726 | b | 3,0495 | 0,2483 | c |
| B05 | 9,0428 | 0,2937 | a | 5,6604 | 0,2689 | b | 2,6130 | 0,2445 | c |
| B013 | 9,3460 | 0,3055 | a | 5,9636 | 0,2800 | b | 2,9163 | 0,2562 | c |
| CR9 | 9,3274 | 0,3055 | a | 5,9450 | 0,2802 | b | 2,8976 | 0,2563 | c |
| CR5 | 8,5284 | 0,2827 | a | 5,1460 | 0,2612 | b | 2,0987 | 0,2385 | c |

For each isolate, LSMean scores obtained from CLMM on the three pea genotypes were significantly different, as shown by different letters between genotypes (Tuckey test, p-value < 0.05) with Lumina, the most susceptible genotype, MN313, the genotype with intermediate behavior and PI180693, the most resistant genotype.
