# Supplementary material for: Genetic and Pathogenicity Diversity of Aphanomyces euteiches Populations From Pea-Growing Regions in France
Source: Front Plant Sci. 2018 Nov 19;9:1673. doi: 10.3389/fpls.2018.01673 (PMC6252352; doi:10.3389/fpls.2018.01673)
Supplement: Supplementary file 4 [file Table_4.DOCX]

**Additional file 9.** LSMeans of disease severity for each genetic cluster of structure analysis without Bourgogne data (delta K=1600)

| **Genetic Cluster** | **Pea** | | | | | | | | | | **Vetch** | | | | | | **Faba Bean** | | | | | **Alfalfa** | | |
| --- | --- | --- | --- | --- | --- | --- | --- | --- | --- | --- | --- | --- | --- | --- | --- | --- | --- | --- | --- | --- | --- | --- | --- | --- |
|  | **Lumina** | | | **MN313** | | | **PI180693** | | | **Amethyste** | | | **Topaze** | | | **Baraca** | | | **Melodie** | | | **Zenith** | | |
|  | LSM | SD |  | LSM | SD |  | LSM | SD |  | LSM | SD |  | LSM | SD |  | LSM | SD |  | LSM | SD |  | LSM | SD |  |
| **1** | 2,264 | 0,293 | a | 2,108 | 0,419 | a | 1,517 | 0,222 | ab | -1,610 | 0,202 | a | -4,630 | 0,576 | a | -0,799 | 0,193 | a | -3,087 | 0,253 | a | -0,363 | 0,191 | a |
| **2** | 2,393 | 0,199 | a | 2,942 | 0,375 | b | 1,460 | 0,171 | a | 3,187 | 0,189 | b | -4,381 | 0,525 | a | -0,078 | 0,135 | b | -2,427 | 0,174 | b | 0,536 | 0,140 | b |
| **3** | 3,250 | 0,230 | b | 2,146 | 0,366 | a | 1,092 | 0,154 | b | 2,749 | 0,169 | c | -4,614 | 0,519 | a | -0,011 | 0,108 | b | -2,685 | 0,160 | ab | 0,459 | 0,119 | b |

For each variety, LSMean scores obtained from CLM followed by the same letter are not significantly different (Tuckey test, p -value > 0.05).
