# Supplementary figures and images for: Genetic and Pathogenicity Diversity of Aphanomyces euteiches Populations From Pea-Growing Regions in France
Source: Front Plant Sci. 2018 Nov 19;9:1673. doi: 10.3389/fpls.2018.01673 (PMC6252352; doi:10.3389/fpls.2018.01673)

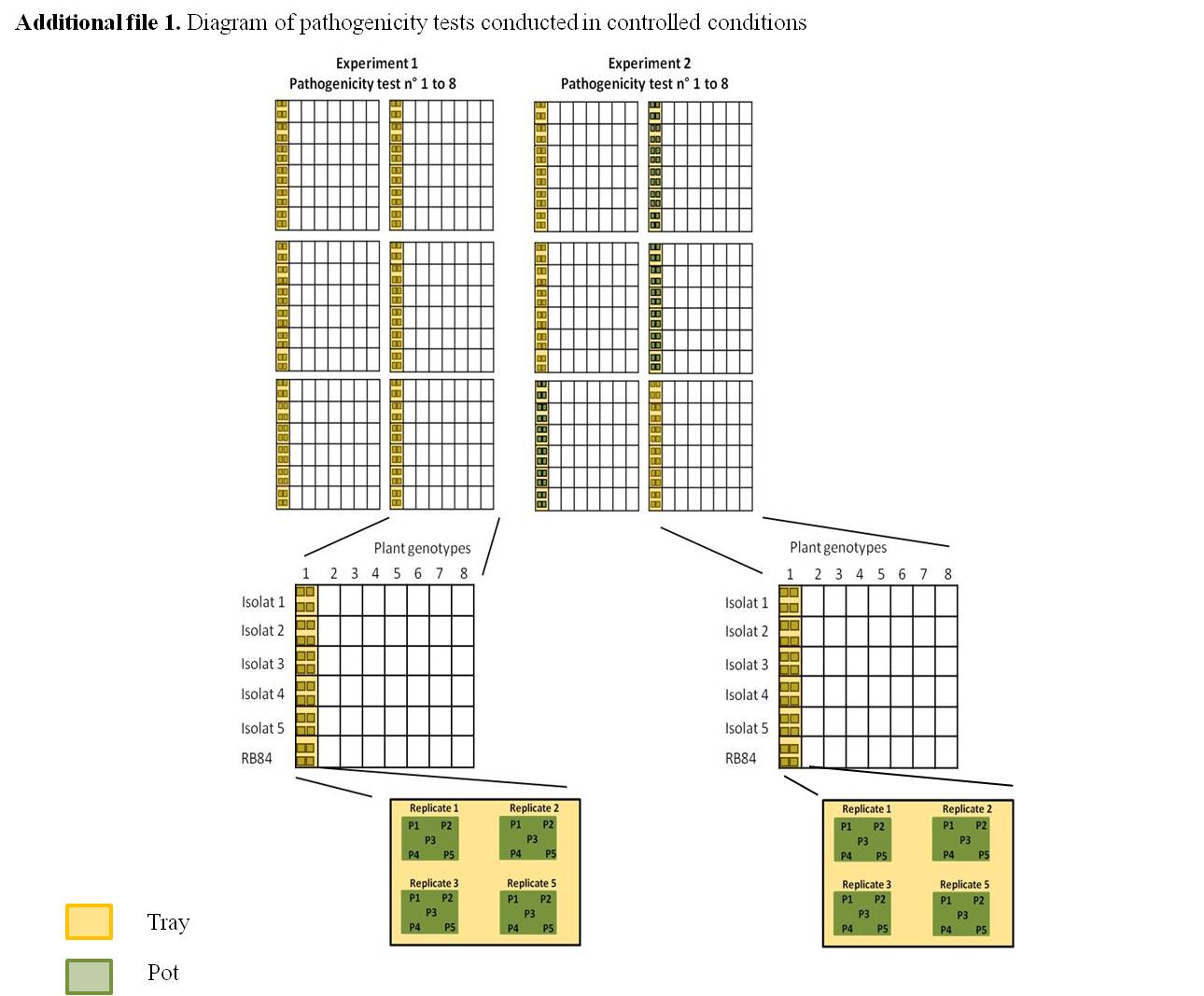

Supplement: Supplementary file 5 [file Image_1.JPEG]

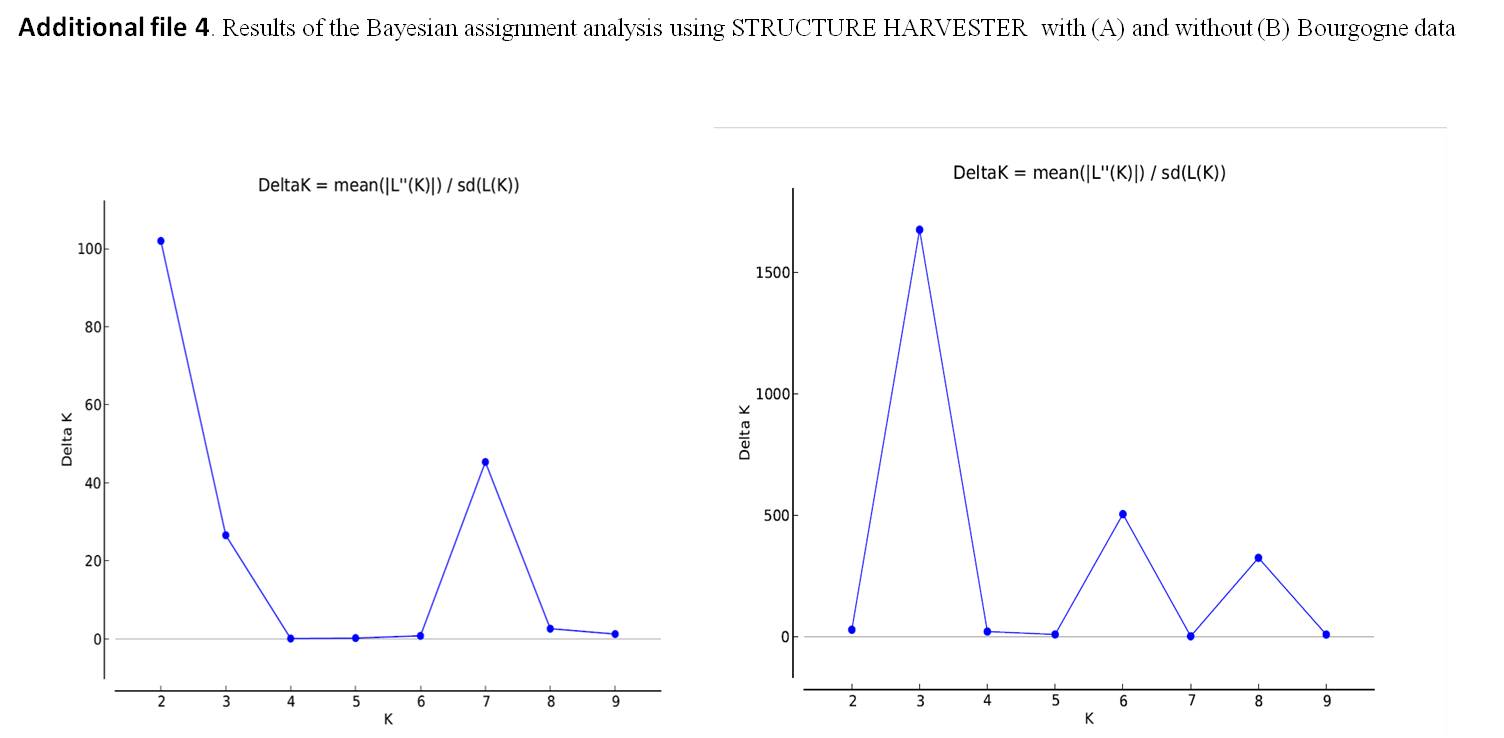

Supplement: Supplementary file 6 [file Image_2.JPEG]

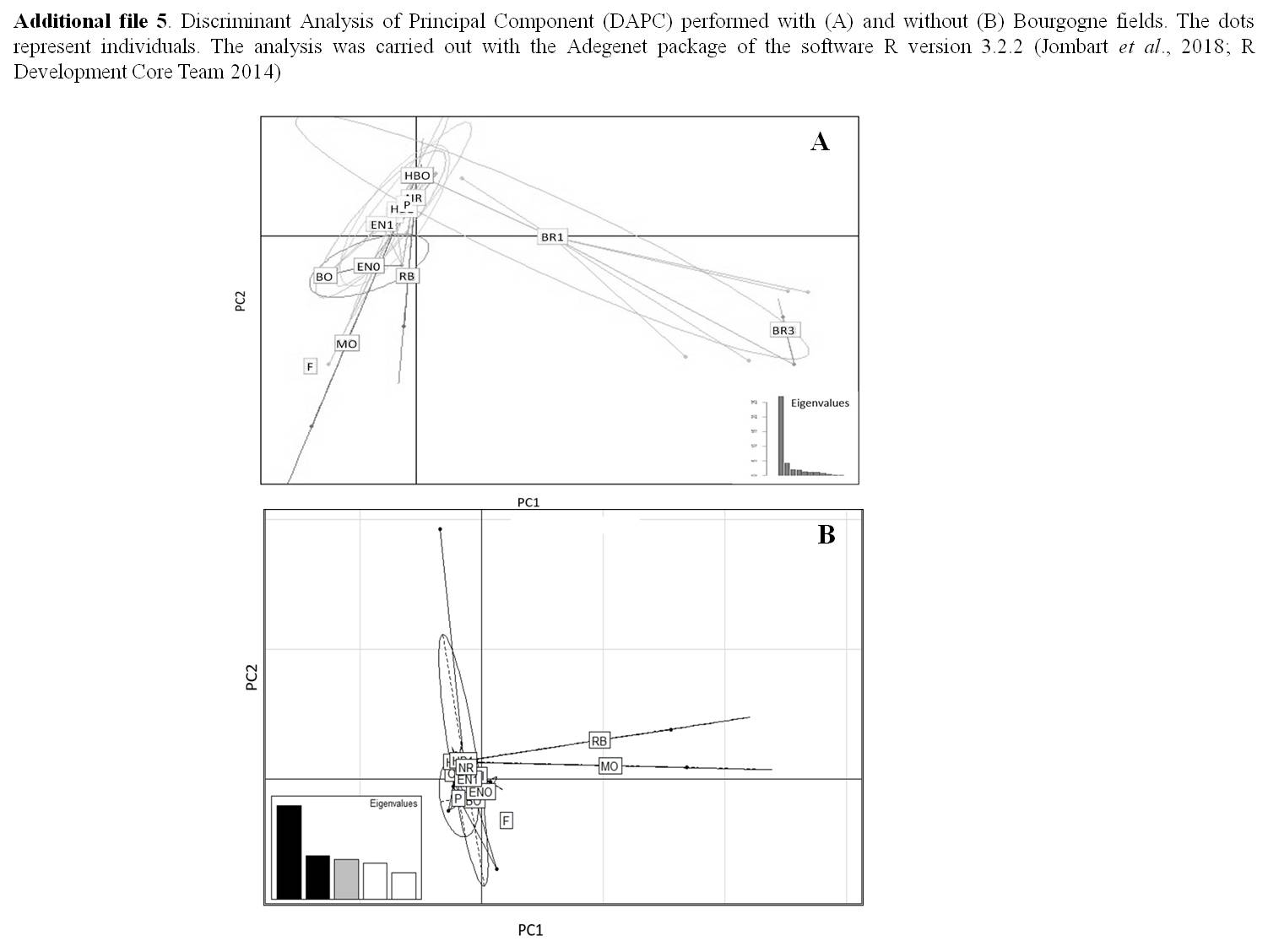

Supplement: Supplementary file 7 [file Image_3.JPEG]

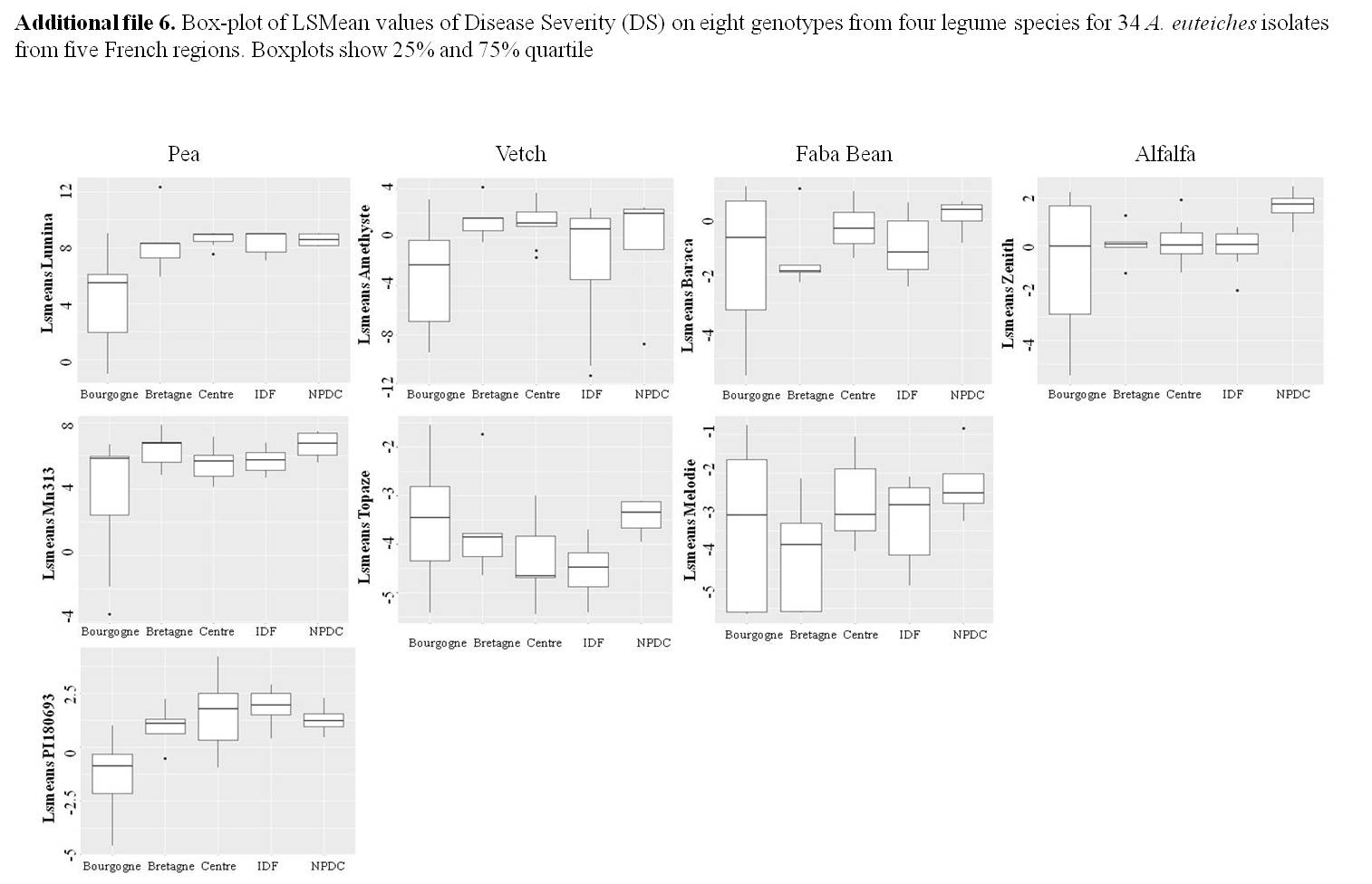

Supplement: Supplementary file 8 [file Image_4.JPEG]

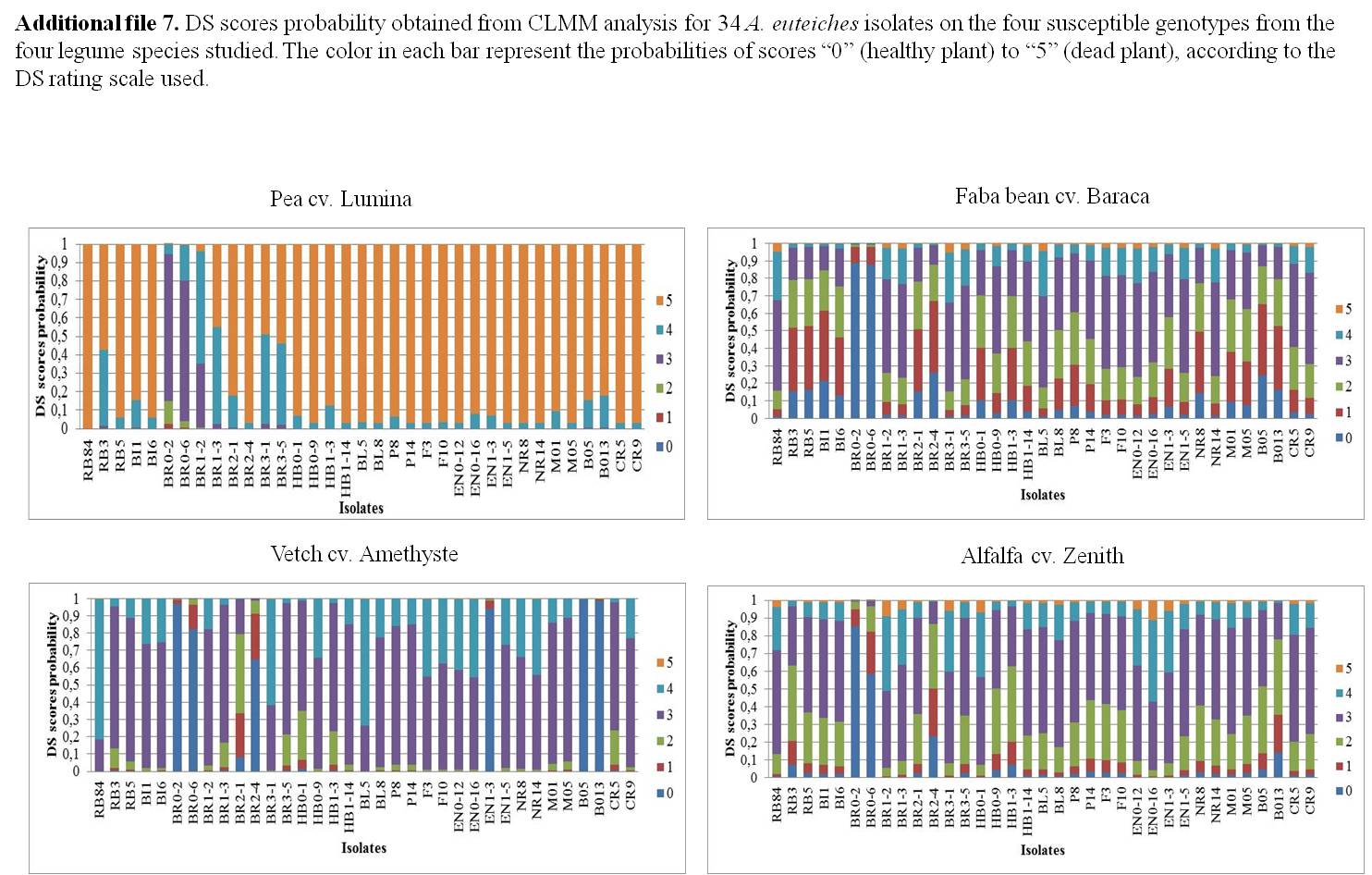

Supplement: Supplementary file 9 [file Image_5.JPEG]
